# Prediction of clinical depression scores and detection of changes in whole-brain using resting-state functional MRI data with partial least squares regression

**DOI:** 10.1371/journal.pone.0179638

**Published:** 2017-07-12

**Authors:** Kosuke Yoshida, Yu Shimizu, Junichiro Yoshimoto, Masahiro Takamura, Go Okada, Yasumasa Okamoto, Shigeto Yamawaki, Kenji Doya

**Affiliations:** 1 Graduate School of Informatics, Kyoto University, Kyoto, Japan; 2 Neural Computation Unit, Okinawa Institute of Science and Technology Graduate University, Okinawa, Japan; 3 Integrated Open System Unit, Okinawa Institute of Science and Technology Graduate University, Okinawa, Japan; 4 Graduate School of Information Science, Nara Institute of Science and Technology, Nara, Japan; 5 Department of Psychiatry and Neurosciences, Hiroshima University, Hiroshima, Japan; National University of Defense Technology College of Mechatronic Engineering and Automation, CHINA

## Abstract

In diagnostic applications of statistical machine learning methods to brain imaging data, common problems include data high-dimensionality and co-linearity, which often cause over-fitting and instability. To overcome these problems, we applied partial least squares (PLS) regression to resting-state functional magnetic resonance imaging (rs-fMRI) data, creating a low-dimensional representation that relates symptoms to brain activity and that predicts clinical measures. Our experimental results, based upon data from clinically depressed patients and healthy controls, demonstrated that PLS and its kernel variants provided significantly better prediction of clinical measures than ordinary linear regression. Subsequent classification using predicted clinical scores distinguished depressed patients from healthy controls with 80% accuracy. Moreover, loading vectors for latent variables enabled us to identify brain regions relevant to depression, including the default mode network, *the right superior frontal gyrus*, and *the superior motor area*.

## Introduction

Advances in analyzing large datasets with machine learning algorithms promote their application in medical diagnosis. In particular, their use in objective diagnosis of psychiatric disorders using brain imaging and other biological data is now being actively studied [1]. A major challenge in applying statistical machine learning algorithms to brain imaging or genetic data is the high dimensionality of the input variables, such as the number of voxels and the number of possible genetic polymorphisms. Even though algorithms such as support vector machine (SVM) and L1-regularized classifiers (LASSO) manage the issue of high-dimensionality, the problem of co-linearity in brain imaging data remains. Neural activities in nearby voxels or in the same functional network are highly correlated, which makes the results of commonly used regression or classification tools unreliable. In this paper, we propose the use of partial least squares (PLS) regression [2–7] with multiple clinical measures to address this problem. We use resting-state functional magnetic resonance imaging (rs-fMRI) data and clinical measures from clinically depressed patients and healthy control subjects to obtain low-dimensional representations of symptoms and brain activities, and we use them to predict depression-related clinical measures and thereafter, to classify subjects.

Use of rs-fMRI is gaining attention in diagnosis of psychiatric disorders because it makes few cognitive demands in measurements and because it can be applied to multiple disorders [8]. In depressed patients, functional connectivities (FCs) between brain areas estimated using rs-fMRI show distributed changes throughout the entire brain [9–12]. Zeng et al. (2012) [13] demonstrated that ∼94% of 53 subjects could be correctly classified as patients or healthy controls using FCs and linear SVM, and they reported that the majority of discriminating FCs were distributed within or across the default mode network, the affective network, visual cortical areas, and the cerebellum. While the aforementioned study sought to discern differences between patients and healthy controls in a binary manner, Zhang et al. (2011) [14] tried to predict clinical measures of the Beck Depression Inventory II (BDI-II) [15] by regressing fMRI signals acquired during a face-watching task. They showed that true and predicted BDI-II were significantly correlated (*r* = 0.55) and using the standard threshold of 14 for the predicted BDI-II, 89% of the automated diagnoses agreed with those of psychiatrists.

Clinical depression is characterized by multiple, related symptoms [16]. There are various clinical measures for assessing symptoms, such as the Snaith-Hamilton Pleasure Scale (SHAPS) [17] for anhedonia and Positive and Negative Affect Schedule (PANAS) [18] for altered mood. In addition, the age of subjects is important for diagnosis since aging increases the risk of depression in general [19].

Here, we consider a two-step approach which predicts multiple measures of clinical depression from rs-fMRI in the first step, and then uses results of the first step for diagnosis. For the first step, we train a regression model to predict BDI-II, SHAPS, PANAS(n), and age from functional connectivity data. Although this could be done using ordinary least squares regression, in order to tackle the issue of high-dimensionality and co-linearity of the input, we explore the use of partial least squares (PLS) regression [2–7], which maps input and output variables to low-dimensional spaces so that the covariance of data in the latent spaces is maximized. We compare the classification performance of the two-step approach using PLS regression with other classification methods. Thereafter, we consider the use of subject age by testing (i) a model with age as a response variable (output-age model), (ii) a model with age as a predictor (input-age model), and (iii) a model that does not consider age (no-age model).

This paper further develops the basic idea presented in [20] to overcome limitations of linear methods and perform objective diagnosis. In section 2, we illustrate the details of rs-fMRI and clinical measures for subjects. Section 3 provides the mathematical basis of PLS and its kernel variants. In addition, it is extended to classification models for the purpose of objective diagnosis. In section 4, we illustrate the efficacy of our application in predicting clinical measures, discriminating between patients and healthy controls, and interpreting derived coefficients. Finally, we offer our conclusions and discuss future work in section 5.

## Data set

This study was approved by the Human Subjects Research Review Committee at the Okinawa Institute of Science of Technology, as well as the Research Ethics Committee of Hiroshima University (permission nr. 172). Written and informed consent was obtained from all subjects participating in the study.

### Subjects

58 patients (age 26–73, average 42.8 ± 11.9, 33 female) with major depression disorder were recruited by the Psychiatry Department of Hiroshima University and collaborating medical institutions, based on the Mini-international neuropsychiatric interview (M.I.N.I [21]), which enables doctors to identify psychiatric disorders, according to the Diagnostic and Statistical Manual of Mental Disorders, Fourth Edition (DSM-IV [22]). As a healthy control group, 65 subjects (ages 20–66, average 34.8 ± 13.0, 28 female) with no history of mental or neurological disease, were recruited via advertisements in local newspapers.

### Clinical measures

The following interview- and questionnaire-based measures are used for determination of disease presence and quantification of the severity of two primary symptoms we wish to predict, namely, anhedonia (loss of motivation, loss of pleasure, etc.) and negative mood (low mood, guilty feelings, suicidal thoughts, etc.).

#### Beck Depression Inventory II (BDI-II)

This measure evaluates the presence and severity of depression based on a self-report questionnaire [15]. Subjects are asked to answer 21 questions about feelings of punishment or guilt, suicidal thoughts, etc. Each answer is scored with a value between 0 and 3, with 3 being the most serious. High scores indicate severe symptoms. The standardized score of ≥14 indicates that a subject is suffering from depression.

#### Snaith-Hamilton Pleasure Scale (SHAPS)

This measure was developed to evaluate the level of anhedonia [17]. Subjects are asked to answer 14 questions about hedonic capacity, with scores between 1 and 4. High scores indicate more severe anhedonia.

#### Positive and Negative Affect Schedule (PANAS)

This widely used measure evaluates positive and negative moods of subjects [18, 23]. In this study, we considered only scores related to negative mood items. This measure is generally known as PANAS(n). Subjects are asked to respond to 10 questions about their moods, with answers between 0 and 5. The sum of all scores indicates the strength of their negative moods. Due to an evaluation issue, one subject’s response could not be assessed, so that score was replaced with the mean of the remaining subjects.

Table 1 summarizes scores exhibited for each measure by each group in our study [20]. Although most patients showed both anhedonia and negative mood, some exhibited only one trait. Correspondingly, the scores of the BDI-II, SHAPS, and PANAS(n) are highly, but not completely correlated. As decreased mental function results from aging, the age of the subjects is expected to correlate with BDI-II, SHAPS, and PANAS(n) as well.

**Table 1:** Mean (± standard deviation) of clinical measures [20].

	Controls	Patients
Number of subjects	65	58
Age	34.8 (±13.0)	42.8 (±11.9)
BDI-II	6.92 (±5.9)	30.9 (±9.0)
SHAPS	23.3 (±6.2)	37.8 (±5.5)
PANAS(n)	8.5 (±6.4)	25.1 (±7.9)

We verified these correlations by calculating the correlation coefficients (Table 2) [20]. Strong correlations between clinical measures are reflected in coefficients above 0.7. Weaker correlations between age and individual clinical measures were around 0.3. In our regression model, BDI-II, SHAPS, PANAS(n), and age of each subject are considered as responses in order to correct for their natural correlation, resulting from functional connectivity. We will show that the introduction of subject age as an output rather than as an input is beneficial with respect to classification accuracy.

**Table 2:** The Pearson’s correlation coefficients between the clinical measures and the subjects’ age [20].

	Age	BDI-II	SHAPS	PANAS(n)
BDI-II	0.2451	-	0.7883	0.8005
SHAPS	0.3221	0.7883	-	0.7497
PANAS(n)	0.2480	0.8005	0.7497	-

### Functional connectivity of resting-state fMRI

Functional MRI measurements were acquired on a 3T GE Signa HDx scanner with a 2D EP/GR (TR = 3s, TE = 27ms, FA = 90deg, matrix size 64x64x32, voxel size 4x4x4 mm, no gap, interleaved). Subjects were instructed to lie with their eyes open, to think of nothing in particular, and to remain awake. They are also instructed to refrain from taking caffeine, nicotine, and alcohol in the day of experiment.

For each subject, acquired images were processed with SPM8 (Wellcome Trust Centre for Neuroimaging, UCL, London) following standard procedures. We first perform slice timing correction, motion correction, co-registration to anatomical MRI, normalization with standard brain and smoothing (Gaussian of full-width at half-maximum 8mm). We confirmed that there were no significant differences in six motion parameters between two diagnostic groups in order to reject a possible effect of spurious functional connectivity due to head motion [24, 25]. Voxels were assigned to 116 brain regions, according to the automatic anatomical labeling atlas (AAL) [26]. Mean activation time series in each brain region were obtained by averaging MRI signal time series over all voxels assigned to each region. Finally, functional connectivity between each pair of regions was computed as the cross correlation of the corresponding time-series.

## Methods

Partial least squares (PLS) regression is a method for modeling a relationship between two sets of multivariate data via a latent space, and of performing least squares regression in that space. PLS can handle high-dimensional co-linear datasets because of its underlying assumption that the two datasets are generated by a small number of latent components. In this process, latent components are formed by maximizing the covariance between the two datasets.

### Partial Least Squares Regression (PLS)

PLS models a linear relationship between two blocks of variables ${\left\{x_{i}\right\}}_{i=1}^{n}\in R^{p}$ and ${\left\{y_{i}\right\}}_{i=1}^{n}\in R^{q}$. In the following parts, *X* = (**x**_1_, …, **x**_*n*_)^*T*^ represents the (*n* × *p*) predictor matrix and *Y* = (**y**_1_, …, **y**_*n*_)^*T*^ represents the (*n* × *q*) response matrix. This procedure obtains *L* latent components as ${\left\{t_{i}\right\}}_{i=1}^{L}$ and ${\left\{u_{i}\right\}}_{i=1}^{L}$. This technique assumes following decomposition:

$$\begin{array}{ccc} X & = & TP^{T}+F_{x} \end{array}$$ (1)

$$\begin{array}{ccc} Y & = & UQ^{T}+F_{y}, \end{array}$$ (2)

where both *T* = (**t**_1_, …, **t**_*L*_) and *U* = (**u**_1_, …, **u**_*L*_) are the (*n* × *L*) matrices of *L* latent components corresponding to *X* and *Y*, respectively. The (*p* × *L*) matrix *P* and the (*q* × *L*) matrix *Q* are loadings and the (*n* × *p*) matrix *F*_*x*_ and the (*n* × *q*) matrix *F*_*y*_ are the matrices of residuals. Since our objective is to perform least squares regression in a low-dimensional latent space, the underlying assumption is that the latent component **u**_*i*_ can be well predicted from **t**_*i*_ from a relation such as:

$$\begin{array}{c} U=TD, \end{array}$$ (3)

where *D* is the (*L* × *L*) matrix. We need to maximize the covariance between **t**_*i*_ and **u**_*i*_ to satisfy the above assumption.

Our objective criterion is

$$\begin{array}{c} \max_{t,u}cov\left(t,u\right)=\max_{w,c}cov\left(Xw,Yc\right), \end{array}$$ (4)

where $w\in R^{p}$ and $c\in R^{q}$ are weight vectors for projection into the latent components.

After extracting the latent component, the observation matrices *X* and *Y* are deflated by subtracting their rank-one approximation. It is important to stress the asymmetry scheme, i.e. that *Y* is deflated based on **t**, in the case of regression. By repeating the above procedures *L* times, we obtain the weight matrices *W* = (**w**_1_, …, **w**_*L*_) and *C* = (**c**_1_, …, **c**_*L*_).

Finally, the relation in the original data space is expressed by

$$\begin{array}{c} Y=XB+E, \end{array}$$ (5)

where *B* is the (*p* × *q*) matrix of regression coefficients and *E* is the (*n* × *q*) matrix of residuals.

Plugging the relationship *B* = *W*(*P*^*T*^*W*)^−1^*C*^*T*^ [27, 28] into Eq (5), we obtain a different representation of *Y* as:

$$\begin{array}{ccc} \hat{Y} & = & XB \end{array}$$ (6)

$$\begin{array}{c} =XW{\left(P^{T}W\right)}^{-1}C^{T} \end{array}$$ (7)

$$\begin{array}{c} =XX^{T}U{\left(T^{T}XX^{T}U\right)}^{-1}T^{T}Y. \end{array}$$ (8)

The final transformation is derived from the following equalities [29],

$$\begin{array}{ccc} W & = & X^{T}U, \end{array}$$ (9)

$$\begin{array}{ccc} P & = & X^{T}T, \end{array}$$ (10)

$$\begin{array}{ccc} C & = & Y^{T}T. \end{array}$$ (11)

Note that $t_{i}^{T}t_{j}=\delta_{ij}$ (the Kronecher delta) takes the values 1 for *i* = *j* and 0 for *i* ≠ *j* as a consequence of the algorithm.

**Fig 1:**
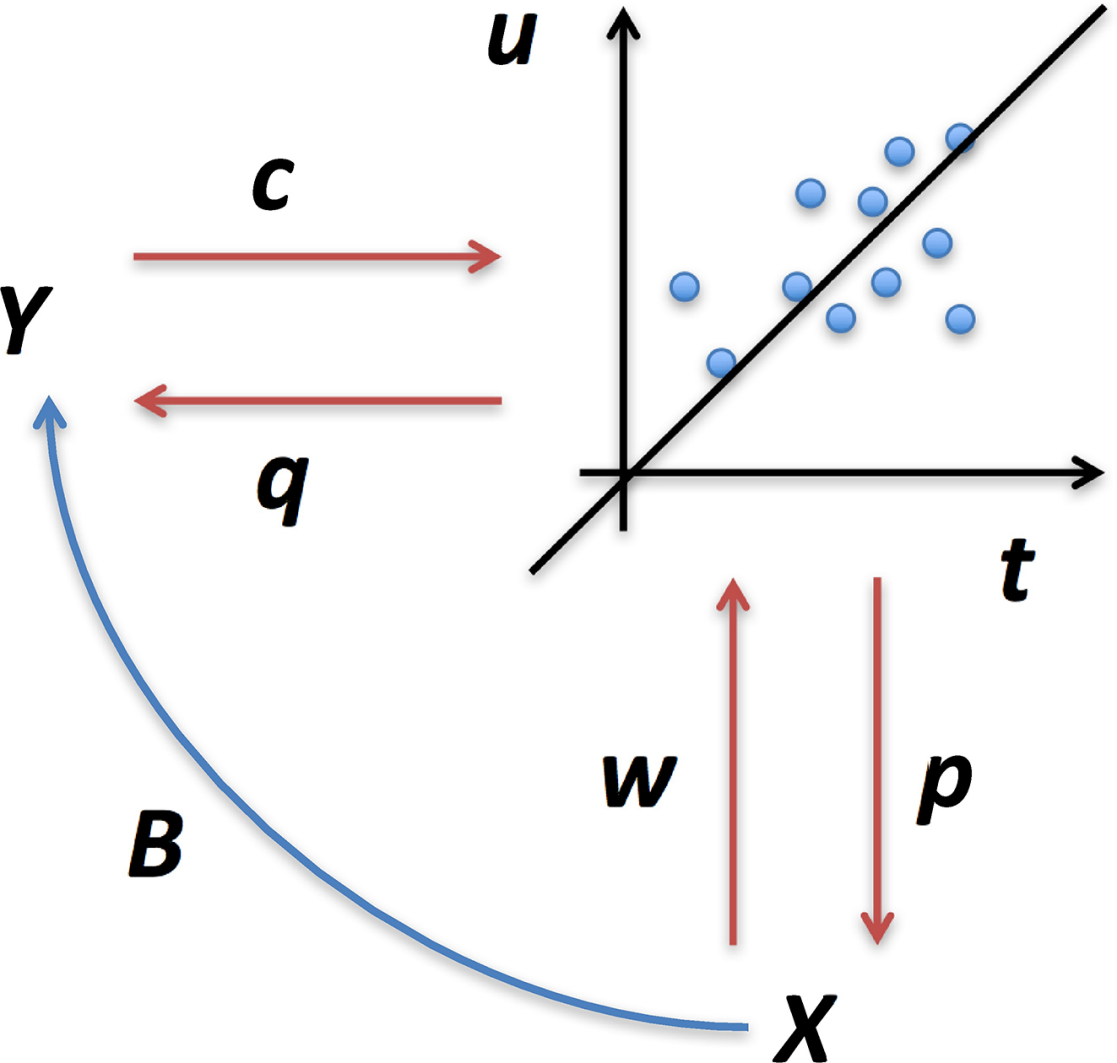
Schematic illustration of partial least squares regression. Two blocks of data, *X* and *Y*, are projected by **w** and **c** onto latent components, **t** and **u**, and least squares regression is performed. **p** and **q** represent loading vectors.

In general, *B* is obtained from a centered training dataset. The response **y**_*new*_ for a new subject **x**_*new*_, referred to as test dataset, is then estimated as follows:

$$\begin{array}{c} y_{new}=\bar{y}+B^{T}\left(x_{new}-\bar{x}\right), \end{array}$$ (12)

where $\bar{y}$ and $\bar{x}$ represent the mean predictor and response in the training dataset, respectively. A schematic outline of PLS is illustrated in Fig 1 and S1 Appendix.

### Kernel Partial Least Squares Regression (KPLS)

Linear PLS is easily extended to nonlinear regression using a kernel trick [28, 30].

Let $\phi :R^{p}\to H$ be a nonlinear transformation of the predictor, $x\in R^{p}$, into a feature vector, ϕ(x)∈H, where H is a high-dimensional feature space. Define a Gram matrix *K* as inner products of points in feature space, i.e., *K* = ΦΦ^*T*^, where Φ = (*ϕ*(**x**_1_), …, *ϕ*(**x**_*n*_))^*T*^ represents the predictor matrix in feature space. In general, the number of columns of Φ is so large that with the explicit form of Φ, we can not perform the same procedure as in the linear case. However, due to the kernel trick, the explicit form of Φ becomes unnecessary.

The deflation procedure is performed as follows:

$$\begin{array}{ccc} K & \leftarrow & \left(I_{n}-tt^{T}\right)K\left(I_{n}-tt^{T}\right) \end{array}$$ (13)

$$\begin{array}{ccc} Y & \leftarrow & Y-tt^{T}Y, \end{array}$$ (14)

where *I*_*n*_ represents an *n*-dimensional identity matrix.

We obtain the prediction on the training data from:

$$\begin{array}{ccc} \hat{Y} & = & \Phi B \end{array}$$ (15)

$$\begin{array}{ccc} & = & \Phi \Phi^{T}U{\left(T^{T}\Phi \Phi^{T}U\right)}^{-1}T^{T}Y \end{array}$$ (16)

$$\begin{array}{ccc} & = & KU{\left(T^{T}KU\right)}^{-1}T^{T}Y. \end{array}$$ (17)

To exclude the bias term, we assume that the responses and the predictors are set to have zero mean in the feature space by applying the following procedure to test kernel *K*_*t*_ and training kernel *K* [31]:

$$\begin{array}{ccc} K_{t} & \leftarrow & \left(K_{t}-\frac{1}{n}1_{n_{t}}1_{n}^{T}K\right)\left(I_{n}-\frac{1}{n}1_{n}1_{n}^{T}\right) \end{array}$$ (18)

$$\begin{array}{ccc} K & \leftarrow & \left(I_{n}-\frac{1}{n}1_{n}1_{n}^{T}\right)K\left(I_{n}-\frac{1}{n}1_{n}1_{n}^{T}\right), \end{array}$$ (19)

where **1**_*n*_ represents the *n*-length vector whose *n* elements are 1. Note that *n* and *n*_*t*_ represent the number of training and test samples, respectively.

In the following section of this paper, we investigate three kernel functions: 1) a second order polynomial kernel *k*(*x*, *x*′) = (*x*^*T*^*x*′ + 1)^2^, referred to as KPLS-Poly(2), 2) a third order polynomial kernel *k*(*x*, *x*′) = (*x*^*T*^*x*′ + 1)^3^, referred to as KPLS-Poly(3), 3) a Gaussian kernel *k*(*x*, *x*′) = exp(−*γ*||*x* − *x*′||)^2^), referred to as KPLS-Gauss, where *γ* is a hyper parameter and set to the inverse of the median of the Euclidian distance of data points.

### Classification

In addition to predicting clinical measures, our aim is to classify subjects into depressed patients and healthy controls using the predicted value of clinical measures for objective diagnosis. We evaluate generalization of binary classifiers using linear discriminant analysis (LDA). Given the training data $D_{tr}=\left\{x_{tr},y_{tr},z_{tr}\right\}$ and test data $D_{te}=\left\{x_{te},y_{te},z_{te}\right\}$, $x\in R^{p}$, $y\in R^{q}$, and **z** ∈ {0, 1} represent functional connectivity as predictors, clinical measures as responses, and binary labels (i.e. 0 is patients and 1 is healthy controls), respectively. In the prediction phase, our objective is to learn the function $f_{B}:R^{p}\to R^{q}$, which, given predictors, **x**_*tr*_, and responses, **y**_*tr*_, assigns predictors to the most probable values of **y**. The prediction on the training dataset is ${\hat{y}}_{tr}=f_{B}\left(x_{tr}\right)$. In the next classification phase, our objective is to learn the classifier $g_{w}:R^{q}\to \left\{0,1\right\}$, which, given predicted responses, ${\hat{y}}_{tr}$, and binary labels, **z**_*tr*_, assigns predicted responses to the most probable labels. Assigned labels on the test dataset are obtained as ${\hat{z}}_{te}=g_{w}\left({\hat{y}}_{te}\right)=g_{w}\left(f_{B}\left({\hat{x}}_{te}\right)\right)$. It is important to stress that the binary classifier is not trained on actual clinical measures, **y**_*tr*_, but on predicted values of ${\hat{y}}_{tr}$.

In a previous study [13], the authors only identified the binary classifier $g_{w}^{{}^{\prime }}:R^{p}\to 0,1$, which, given functional connectivity, **x**_*tr*_, and binary labels, **z**_*tr*_, assigns functional connectivity directly to binary labels. By exploiting the predicted result of clinical measures, it may be possible to improve classification performance. We compared two scenarios, i.e. i) classification of patients and healthy controls using LDA from predicted clinical measures with KPLS (with KPLS-Gauss, KPLS-Poly(3), and KPLS-Poly(2)), PLS, and ordinary least squares regression (OLS), ii) classification of patients and healthy controls by means of LDA and SVM from functional connectivity directly. Note that we perform feature selection before scenario 2) by calculating connection-wise t-tests to determine the connections with different group means, represented by t-scores. We select the *M* functional connections with the highest absolute t-scores. *M* is optimized by cross validations.

### Pre-screening

Even though PLS can cope with high-dimensional, co-linear datasets, we prescreened variables depending on their relevance to responses in the following way.

Based on Pearson correlation coefficients, *ρ*_*rl*_, between the *r*-th functional connection and the *l*-th clinical measures, we define the empirical relevance of the *r*-th functional connection as

$$\begin{array}{c} R_{r}=\sum_{l=1}^{4}\rho_{rl}^{2},\;r=1,\ldots ,p, \end{array}$$ (20)

where *p* is the total number of functional connections.

These functional connections are ranked according to their empirical relevance, ${\left\{R_{r}\right\}}_{r=1}^{p}$, and only *M* relevant functional connections are used in following procedure. The optimal number for M was determined through nested leave-one-out cross-validation.

### Nested leave-one-out cross validation

Conventionally, cross validation is employed to assure generalization ability of a model or to evaluate optimal parameters. Since we have to account for both generalization ability and parameter optimization, we made use of nested leave-one-out cross validation (LOOCV), which consisted of outer and inner LOOCV. The outer LOOCV repeats iterations that split the whole set of samples into a single outer validation sample used to evaluate the generalization ability, and an outer training set for model estimation. The inner loop of LOOCV is performed on the outer training set to optimize two parameters, *M* and *L*, the number of selected predictor variables and the number of components, respectively. The pair of parameters that achieves the lowest root mean squared error based on the inner validation sample are adopted as optimal parameters and used to evaluate the model using the outer LOOCV. These steps are repeated until each sample has served as the validation sample.

### Age

Age is significantly correlated with three clinical measures (Table 2). In general, age matching performed on different diagnostic groups reduces sample size, causing poor performance. To avoid this problem, we investigated three models, i.e. (i) a model with age as a response (denoted by output-age), (ii) a model with age as a predictor (denoted by input-age), and (iii) a model without age (denoted by no-age). By incorporating age into our model, we can cope with age differences among subjects and can fairly evaluate prediction performance.

### Interpretation

Interpretation of each latent component projected from input and output data gives novel insights into the relationship between functional connectivity and clinical measures. In the framework of PLS, loading matrices, *P* and *C*, indicate contributions from predictor variables and response variables to each latent component (see Eqs (10) and (11)). The (*i*, *j*)-element of the loading matrix, *P*, represents the contribution of the *i*-th functional connection to the *j*-th latent component. Similarly, the (*i*, *j*)-element of the loading matrix, *C*, represents the contribution of the *i*-th clinical measure to the *j*-th latent component. Note that due to subject variability, values of *P*_*ij*_ and *C*_*ij*_ vary depending on the training set used.

## Results

### Regression performance

**Fig 2:**
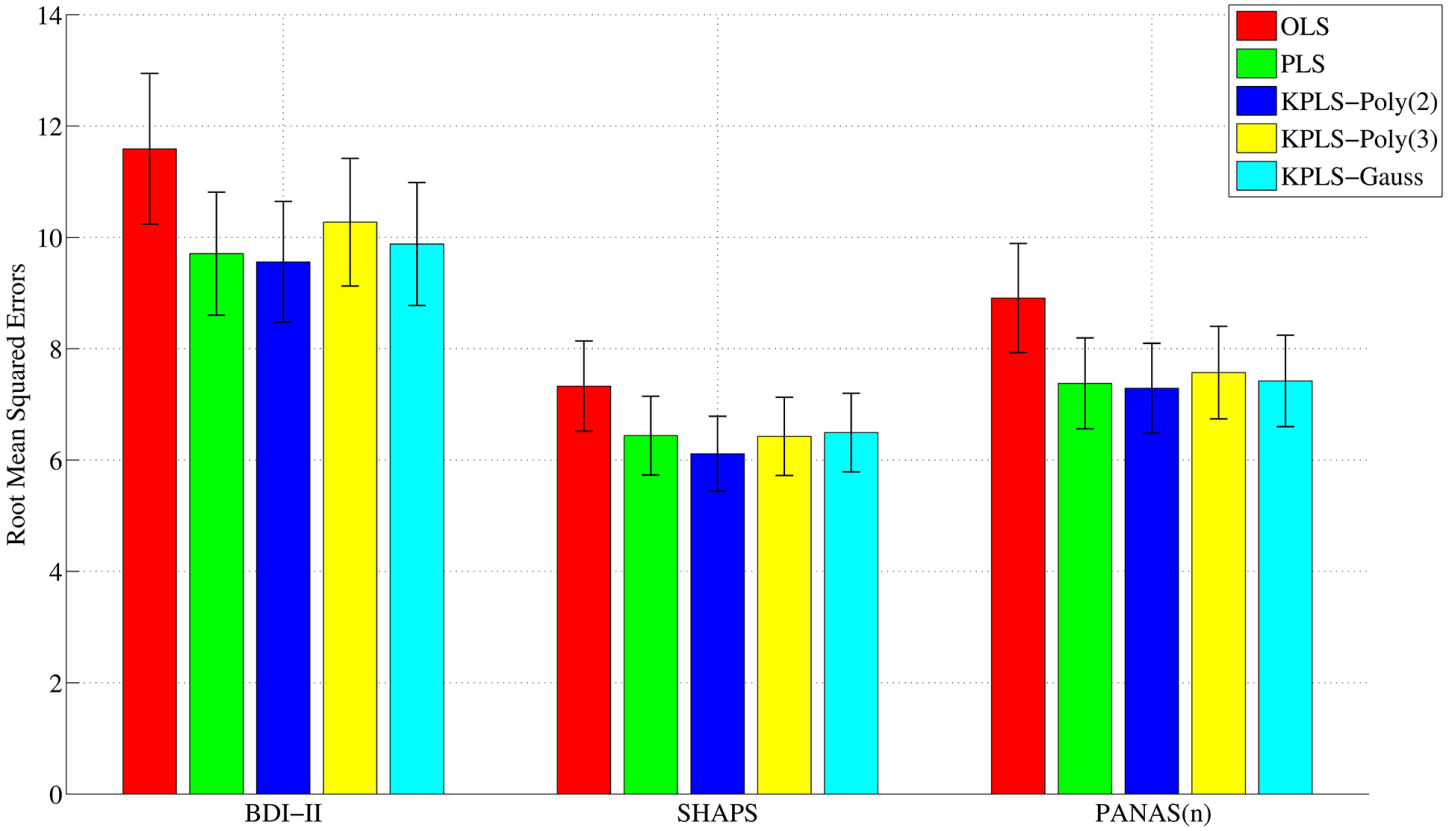
Comparison of predicted performance by means of the root mean squared errors. Linear and kernel variants of PLS achieved significantly better performance than did OLS in all clinical scores. Subject age was used as the output along with clinical scores (output-age model).

We compared the prediction performance of PLS, its kernel variants, and other methods by means of the root mean squared error (RMSE) of the predicted clinical measures in nested leave-one-out cross validation (see Methods). Kernel PLS with a second-order polynomial kernel (KPLS-Poly(2)) achieved the lowest RMSE (9.56 for BDI-II, 6.11 for SHAPS, and 7.29 for PANAS(n)) (Fig 2). This performance was significantly better than that of ordinary least squares regression (OLS) (11.6 for BDI-II, 7.33 for SHAPS, and 8.91 for PANAS(n)) and comparable to that of other variants of PLS applied in our study, suggesting that projection of data into a low-dimensional space was beneficial to regression performance. All statistical comparisons were adjusted for multiplicity using the Bonferroni-Holm method with significance level, *α* = 0.05.

**Fig 3:**
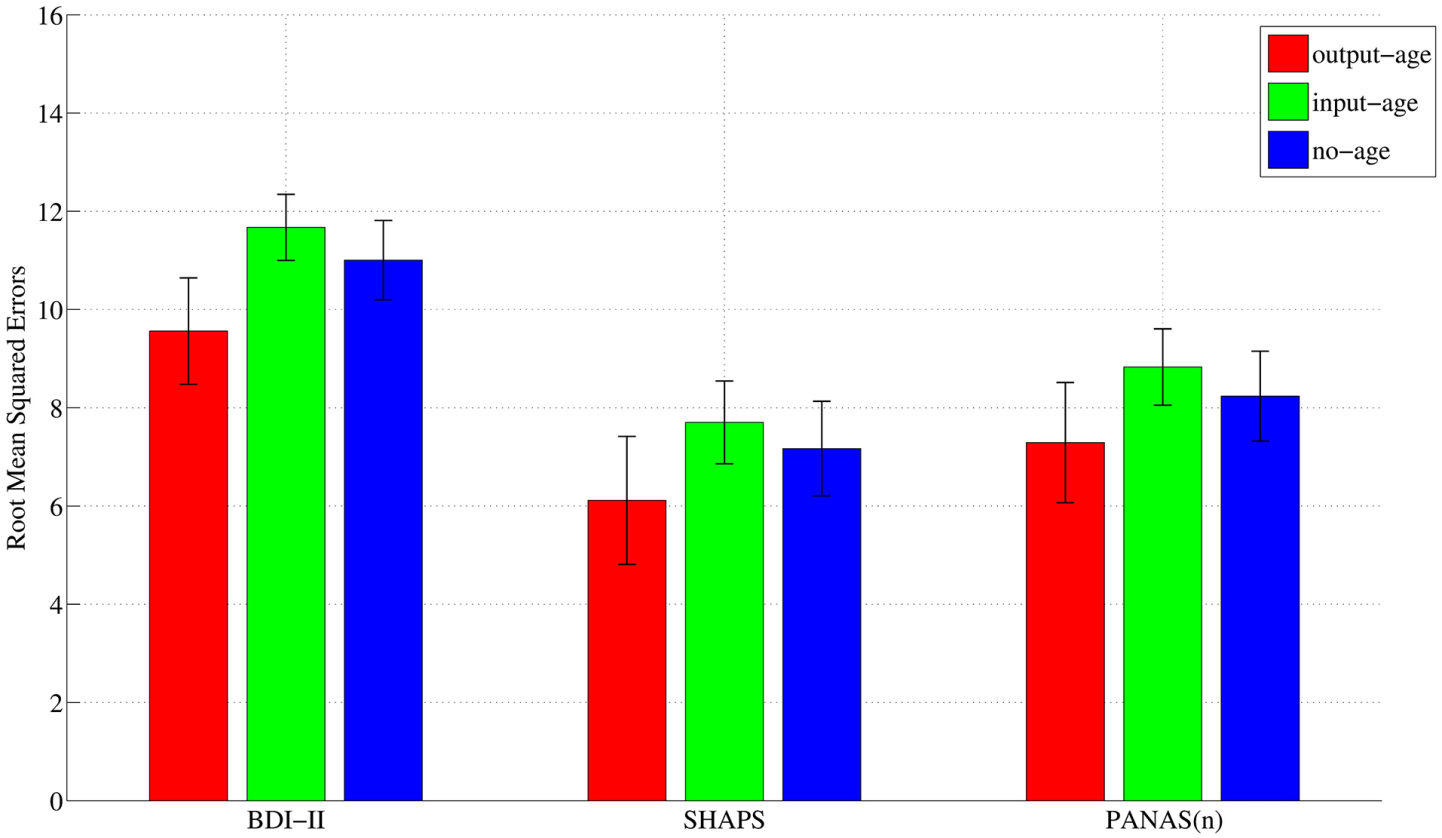
Root mean squared errors in KPLS-Poly(2). KPLS-Poly(2) achieved significantly better performance in output-age model than in other models.

Next, to evaluate the best way of incorporating age into our regression models, we compared RMSE of the output-age, input-age, and no-age models. In our study, incorporating age into our regression model as a response (output-age) achieved significantly better performance than that of the input-age and no-age models (Fig 3). The details were listed in Supporting Information (S1, S2 and S3 Tables). All statistical comparisons were adjusted for multiplicity using the Bonferroni-Holm method with significance level, *α* = 0.05.

**Fig 4:**
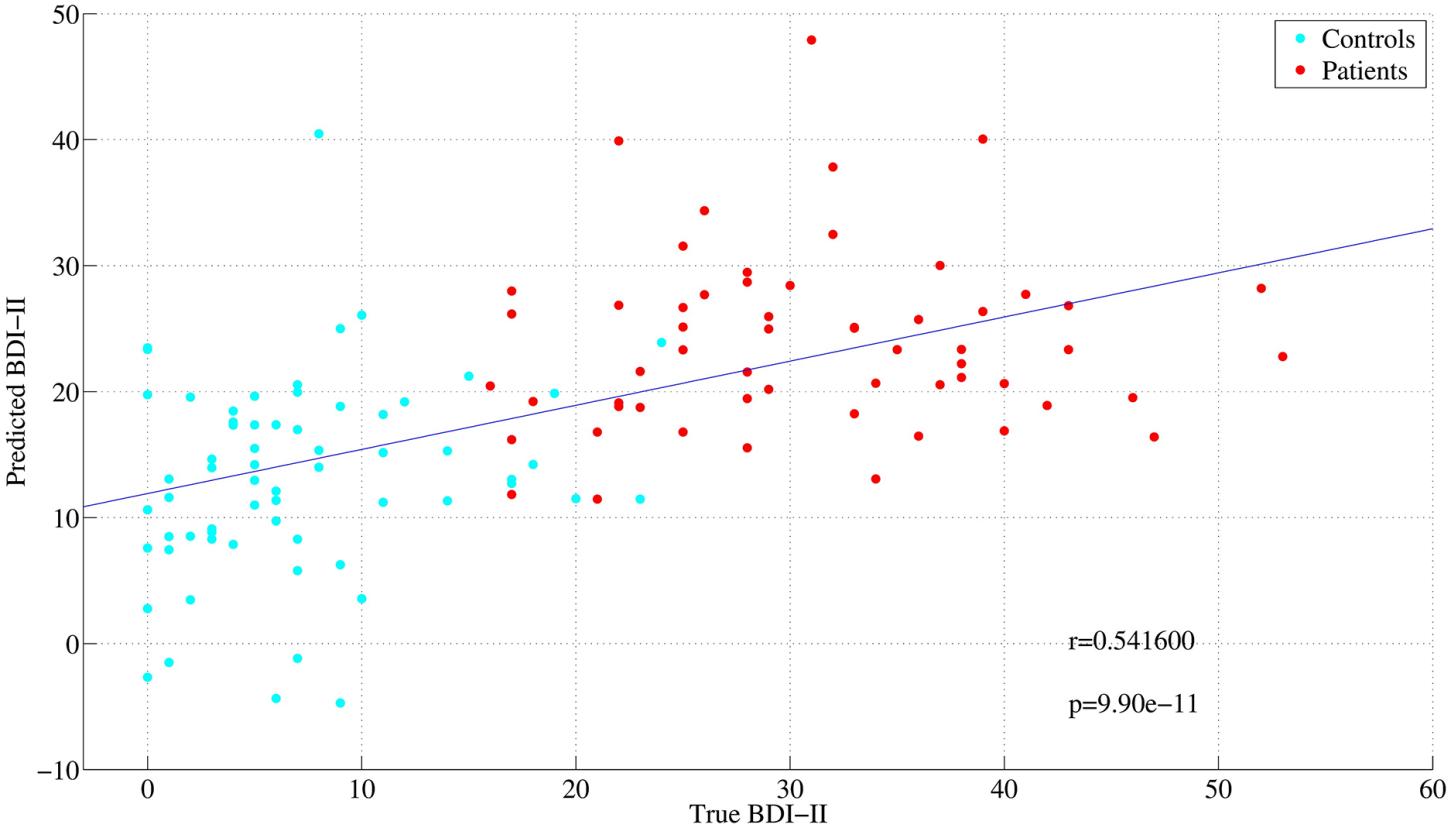
Actual and predicted values of BDI-II. BDI-II were well predicted by KPLS-Poly(2) with RMSE = 9.56 and *r* = 0.541 (*p* < 10^−10^). Red and blue points represent patients and healthy controls, respectively.

The correlation coefficient of actual and predicted values for BDI-II, SHAPS, and PANAS(n) in the case of KPLS-Poly(2) were *r* = 0.541, 0.591, 0.563, respectively. The relationship between predicted and actual values of BDI-II for KPLS-Poly(2) was exemplified (Fig 4). This result was comparable to that of Zhang et al. (2011) [14]; however, the number of subjects in our study was larger than in theirs, reconfirming validity of the results.

The optimal number of retained features M* identified by pre-screening and using the latent component L* identified with nested LOOCV were 40 and 3, respectively, suggesting that reduction of feature size was relevant for improvement of PLS prediction accuracy.

### Classification performance

**Fig 5:**
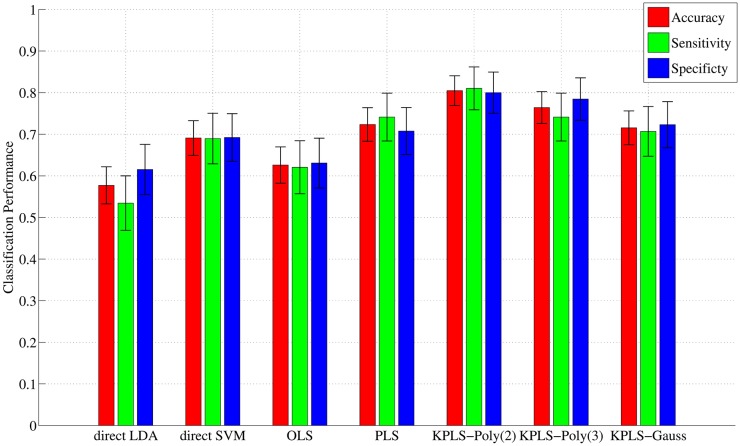
Classification accuracy, sensitivity, and specificity. KPLS-Poly(2) followed by LDA achieves the best performance (accuracy = 80.5%, sensitivity = 81.0%, and specificity = 80.0%).

Projecting the original data onto a low-dimensional space was expected to improve classification accuracy. To verify the benefit of projection, several classification methods were performed and evaluated using accuracy, sensitivity, and specificity (Fig 5). The details were listed in Supporting Information (S4 and S5 Tables). In our study, KPLS-Poly(2) followed by LDA achieved the best accuracy 80.5% (sensitivity 81.0% and specificity 80.0%), which is significantly better than the 57.7% accuracy of direct LDA (sensitivity 53.4%, and specificity 61.5%) and 69.1% accuracy of direct SVM (sensitivity 69.0%, and specificity 69.2%). This result indicates that it was beneficial to exploit the prediction model for clinical measures in order to build a classification model. In addition, KPLS-Poly(2) followed by LDA also achieved significantly better accuracy than the 62.6% accuracy of OLS followed by LDA (sensitivity 62.1% and specificity 63.1%), indicating that considering a latent space in a regression model was beneficial to final classification. Accuracy did not differ significantly between PLS and kernel variants. All statistical tests were based on approximation with the normal and adjusted for multiplicity using the Bonferroni-Holm method with significance level *α* = 0.05.

### Interpretation

**Fig 6:**
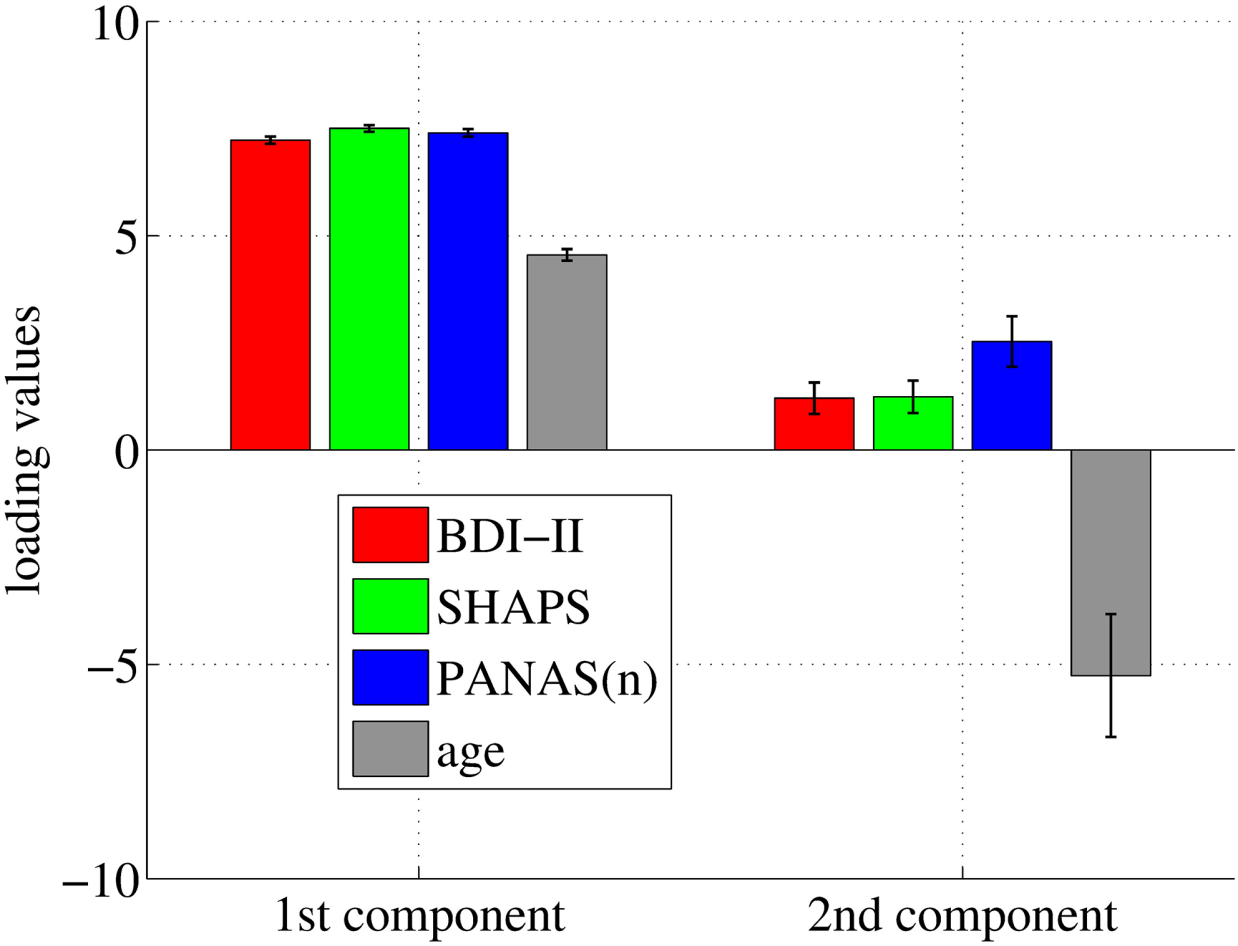
Loading matrix *C*. The matrix indicates contributions of each clinical measure to the first two latent components. Age has a negative influence on the second latent component. Error bars indicate the standard deviation.

In our study, three clinical scores showed almost equally positive influences on the first component, and age also had a positive influence as well. However, age showed a strong negative influence on the second component, in contrast to the clinical scores (Fig 6).

**Fig 7:**
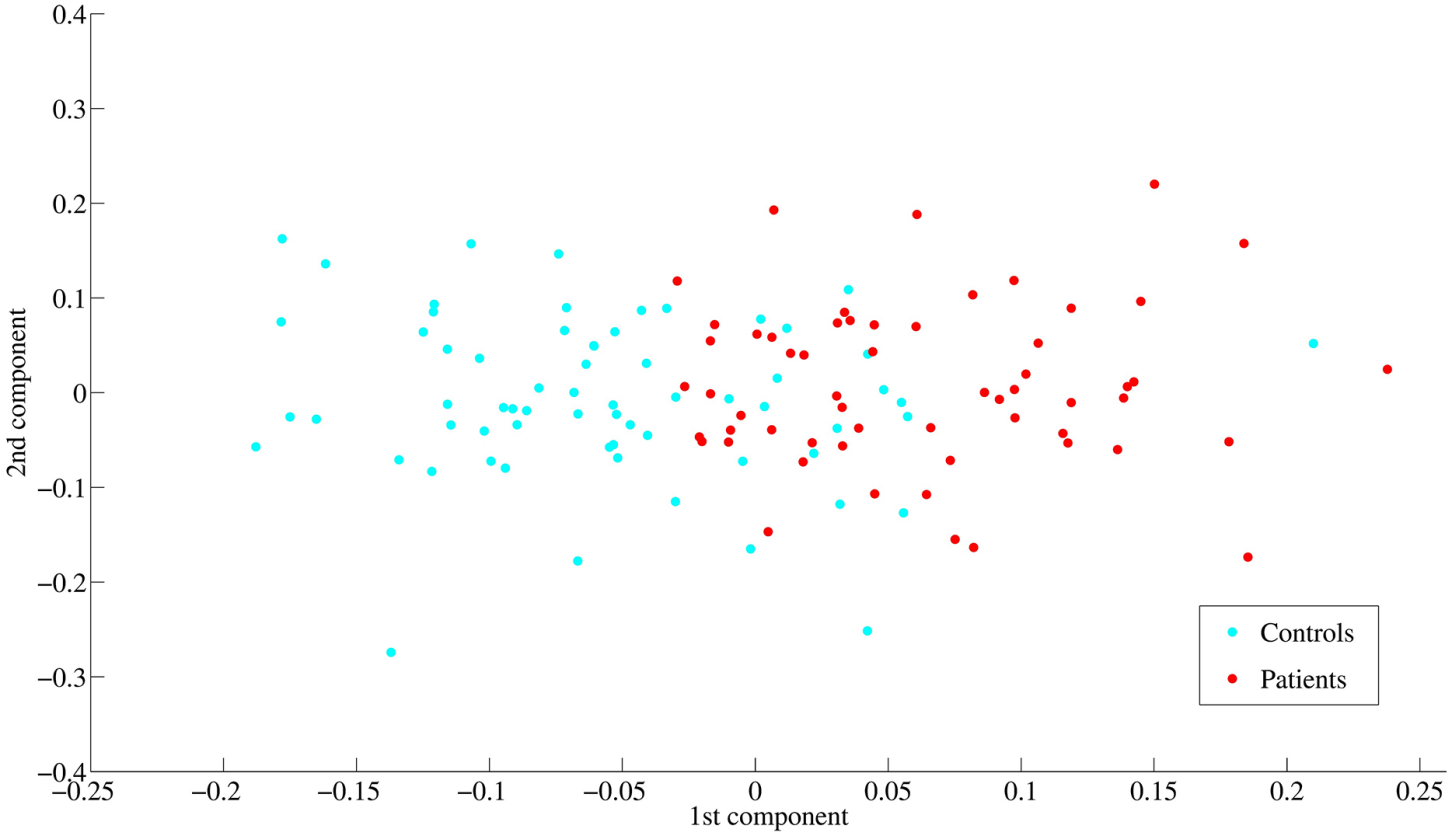
Scatter plot of the latent variables in the first two latent components generated from KPLS-Poly(2). Red and blue dots represent patients and healthy controls, respectively. The two groups are separated mainly by the first latent component.

Latent space representation of subjects showed that the first component explained most depression severity in comparison with the second component (Fig 7). This is consistent with the results of loading matrix *C*. Note that since the optimal number of latent components, in terms of minimizing regression error, was 3, the second and the third components are thought to contain some information about scores.

**Fig 8:**
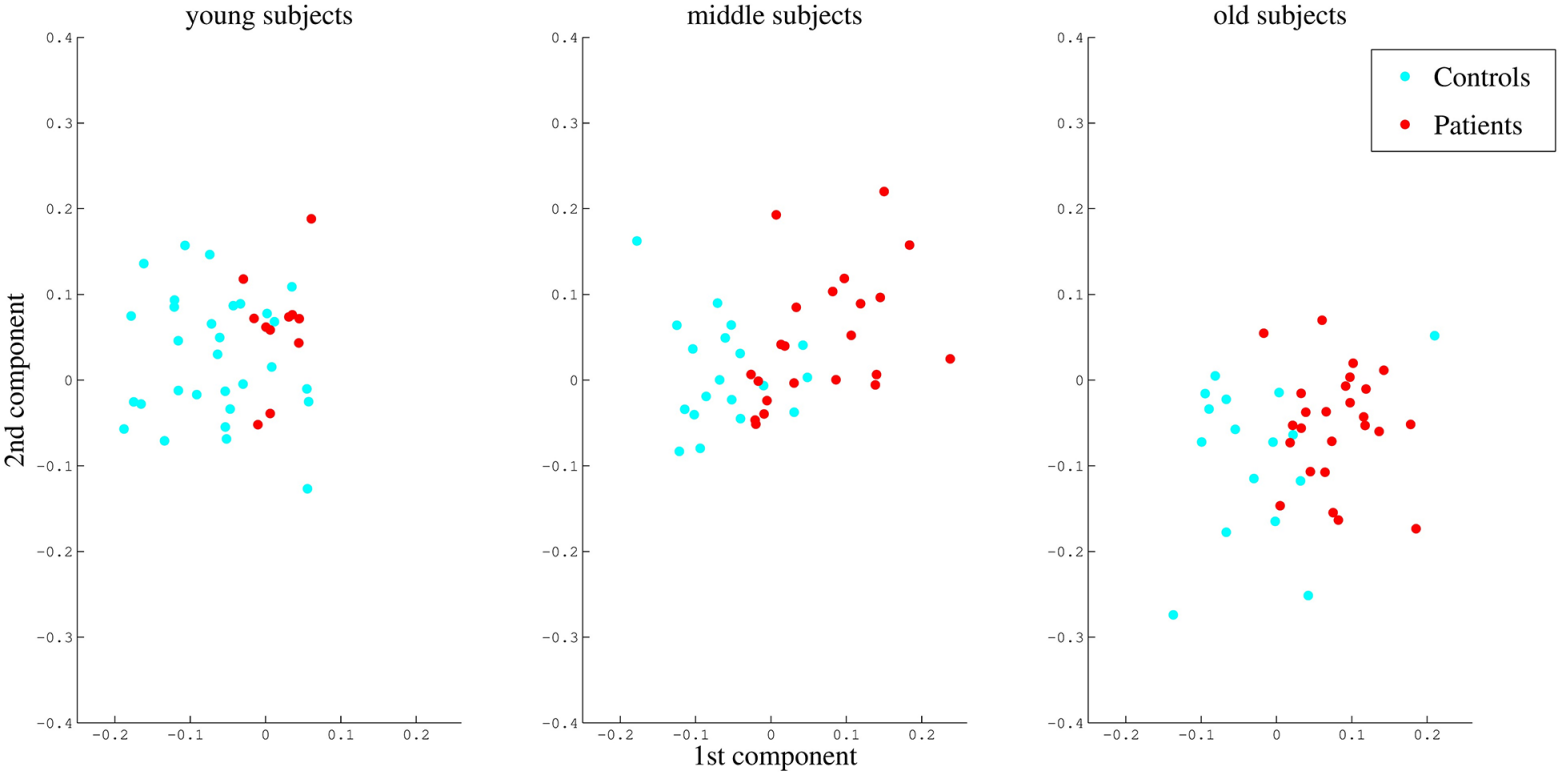
Scatter plot of subjects separated on the basis of the two latent components generated from KPLS-Poly(2) for young, middle, and old subjects. Old subjects have significantly lower values in the second component (*p* < 10^−5^ by Wilcoxon Rank-Sum Test).

In order to validate the effect of age, especially in the second component, all subjects were grouped into young (age 20–31, 41 subjects), middle (age 31–43, 41 subjects), and old (age 44–73, 41 subjects) groups. Note we simply divided the subjects in three equal-sized groups for convenience, “young”, “middle”, and “old”. They are relative, not absolute age classes. Latent variables of old subjects in the second component were significantly lower than those of young and middle subjects (*p* < 10^−5^ by Wilcoxon Rank-Sum Test), suggesting that old patients have distinctive patterns in the second latent space [5] (Fig 8).

Evaluation of loading matrix, *P*, reveals functional connections relevant to each latent component. Especially, the first column of *P*, corresponding to the first component responsible for discrimination of each diagnostic group, is expected to reveal useful insights about the effect of functional connections on depression symptoms. Even though the performance of KPLS-Poly(2) in prediction and classification was comparable to or better than that of linear PLS, patterns of significant loadings were consistent in our experiments. For reasons of interpretation, we therefore focus on the loading matrix of the linear terms in following sections.

**Fig 9:**
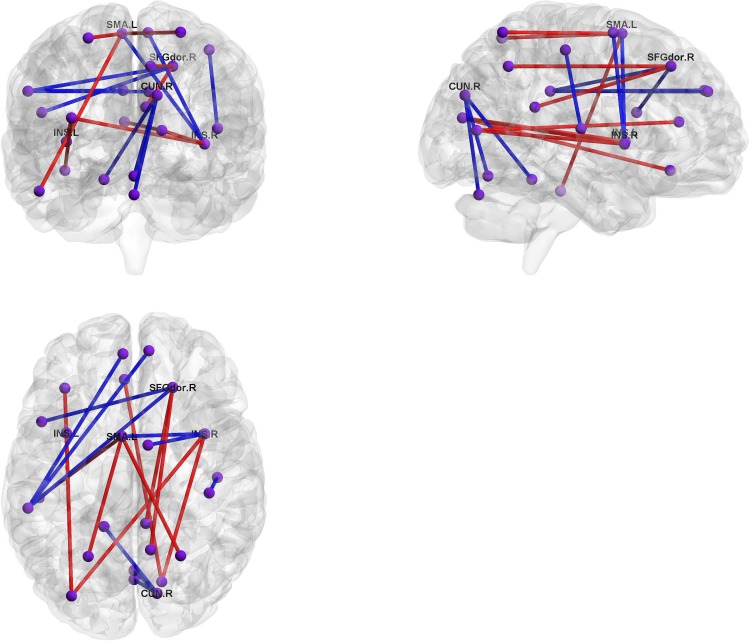
Contributing functional connectivity in first latent component. Red and blue lines represent positive and negative loadings, respectively. SFGdor.R: *right superior frontal gyrus*, INS: *insula*, SMA.L: *left supplementary motor area*, CUN.R:*right cuneus*.

**Fig 10:**
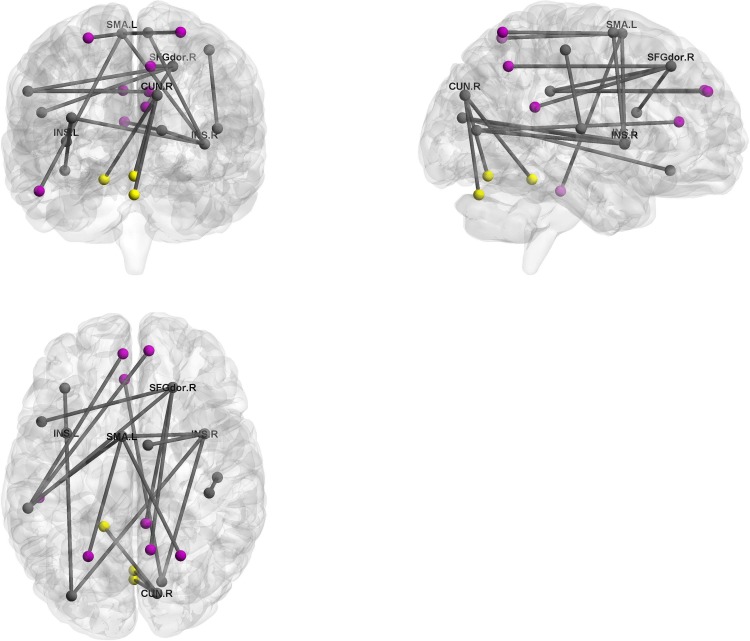
Contributing functional connectivity in first latent component. Purple and yellow nodes represent brain areas within the default mode network and *cerebellum*, respectively. SFGdor.R: *right superior frontal gyrus*, INS: *insula*, SMA.L: *left supplementary motor area*, CUN.R:*right cuneus*.

BrainNet Viewer [32] (http://www.nitrc.org/projects/bnv/) was used to visualize the top 10 connections with positive and negative loadings for the first component (Figs 9 and 10). In this figure, many regions involved in the default mode network (DMN), as well as *the left supplementary motor area*, *the right superior frontal gyrus*, and *the insula*, were relevant. In addition, some functional connectivity between *the right cuneus* and regions involved in *the cerebellum* were negatively correlated with the first component.

## Discussion

MacIntosh et al. (1996) first introduced PLS analysis into the field of neuroimaging in order to extract common information between brain activity and exogenous information, such as experimental or behavioral data [3, 4]. In particular, behavioral data are increasingly used to extract associated brain activity patterns for various types of psychological diseases, such as Alzheimer’s disease [33], obsessive-compulsive disorder [34], and schizophrenia [35]. In these studies, neuropsychological test scores are used as behavioral data, in addition to the labels that represent diagnostic groups and age. To the best of our knowledge, this is the first study to investigate associations between functional connectivity in the whole brain and multiple clinical measures for depressed patients, using PLS and its kernel variants.

Diagnosis based on resting-state functional connectivity is a challenging task due to the high-dimensionality and co-linearity of data. Recent studies have demonstrated that depressed patients can be distinguished from healthy controls by means of their functional connectivity by applying conventional methods, such as support vector machine [9, 13]. Since binary labels are ultimately abstracted information about depression that ignores the severity of symptoms, it is worth considering more detailed information, such as BDI-II, SHAPS, and PANAS(n) to build more sophisticated models. Our study demonstrated that projecting functional connectivity data into a low-dimensional latent space, can predict clinical measures, and can also improve depression diagnostic accuracy.

To separately identify neural circuits associated with anhedonia and negative mood is a challenging task. A psychopathological study suggests that these primary symptoms result from different neural circuits and from alternation of different neurotransmitters [16]. Our results show that SHAPS and PANAS(n) are highly correlated and contributed quite similarly to each latent component (Fig 6), suggesting that further investigation and different approaches may be required to support psychopathological studies from the point of data driven analysis.

We also evaluated extended AAL generated by subdividing standard AAL into 600 regions to examine if the finer atlas could be used to improve the prediction of the clinical scores [36]. The performance was significantly worse than that of standard AAL (S6 Table) and the selected functional connections were inconsistent. Since analysis of brain imaging data with limited sample size highly depends on the choice of ROI, the finer atlas does not necessarily provide better prediction performance. Therefore, it is fare to note that further research is required to validate the best atlas.

### Contributing brain regions

Identification of relevant brain regions in functional connectivity analysis yielded the following three observations: (1) connections between the default mode network and other regions, such as *the right superior frontal gyrus* and *the left supplementary motor area* are relevant (2) *the left and right insulas* in both hemispheres are relevant, (3) connections between *the cerebellum* and *the right cuneus* are relevant.

First, the default mode network (DMN) shows synchronized deactivation during cognitive tasks and is thought to be related to major depressive disorder [37–40]. Our study supports these results, indicating that many relevant connections are related to the DMN, such as *the right posterior cingulum*, *the right precuneus*, and *the superior parietal gyrus*. The DMN contributes positive connections with *the right superior frontal gyrus* and *the left supplementary motor area*. *The superior frontal gyrus*, as a critical region in cognitive tasks, was previously reported to be associated with depression [41]. While *the supplementary motor area* is known to be responsible for motor control, it is also reportedly related to some subtype of depression [42]. Our results support these results.

Second, our results suggest that *the insula* is associated with depression. Some meta-analysis of PET and fMRI studies revealed that *the insula* plays an important role in regulation of emotion [43, 44]. Similarly, resting-state fMRI studies have indicated that *the insula* is directly associated with depression [45, 46].

Finally, we showed that three connections between *the right cuneus*, located in the visual cortical area, and *the cerebellum*, negatively influence depression. While visual processing is believed not to be affected in depression, some previous studies have suggested that it is associated with bipolar disorder [47]. Moreover, regional homogeneity (ReHo) interpreted as a measure of localized synchrony in resting-state fMRI was decreased [48]. *The cerebellum* is usually considered to be responsible for motion control, but our results indicate that it may also be involved in regulation of mood and cognitive processing associated with symptoms of depression. Some fMRI studies demonstrate that this area is responsible for various types of information processing [49, 50], and resting-state functional connectivity studies imply that *the cerebellum* may be critical for the distinction between depressed patients and healthy controls [13, 51].

### Conclusion

In summary, we employed partial least squares regression and its kernel variants to predict clinical measures of subjects using resting-state functional connectivity. Diagnosis of depression based on predicted clinical scores performed better than classification algorithms attempting diagnoses directly from functional connectivity. Moreover, analysis of latent variables identified functional networks relevant to the diagnosis of depression. These results suggest that a low-dimensional representation derived using PLS is beneficial for objective diagnosis. Further investigations are required to separate the two neural circuits associated with two primary symptoms, anhedonia and negative mood.

## Supporting information

S1 Appendix Partial least squares regression. (PDF)

S1 Table Root mean squared errors in output-age. Bold figures represent the best achievement.
(PDF)

S2 Table Root mean squared errors in input-age. (PDF)

S3 Table Root mean squared errors in no-age. (PDF)

S4 Table Classification performance. KPLS-Poly(2) followed by LDA significantly outperformed direct LDA, SVM, and OLS followed by LDA in accuracy (adjusted for multiplicity using the Bonferroni-Holm method with significance level *α* = 0.05).
(PDF)

S5 Table Classification performance without subjects over 60. KPLS-Poly(2) followed by LDA significantly outperformed direct LDA and OLS followed by LDA in accuracy (adjusted for multiplicity using the Bonferroni-Holm method with significance level *α* = 0.05).
(PDF)

S6 Table Root mean squared errors in extended AAL. Performance with the standard AAL outperformed extended AAL significantly. (adjusted for multiplicity using the Bonferroni-Holm method with significance level *α* = 0.05).
(PDF)
