# Supplementary material for: Prediction of clinical depression scores and detection of changes in whole-brain using resting-state functional MRI data with partial least squares regression
Source: PLoS One. 2017 Jul 12;12(7):e0179638. doi: 10.1371/journal.pone.0179638 (PMC5507488; doi:10.1371/journal.pone.0179638)
Supplement: S1 Appendix — (PDF) [file pone.0179638.s001.pdf]

Supporting Information

S1 Appendix. Partial Least Squares Regression.

Algorithm 1 NIPALS procedure

Input: $X_1 = X$ and $Y_1 = Y$
randomly initialize $\mathbf{u}_1$
for $i = 1$ to L **do**
  repeat
    $\mathbf{w}_i = X_i^T \mathbf{u}_i$
    $\mathbf{t}_i = X_i \mathbf{w}_i / \|X_i \mathbf{w}_i\|_2$
    $\mathbf{c}_i = Y_i^T \mathbf{t}_i$
    $\mathbf{u}_i = Y_i \mathbf{c}_i / \|Y_i \mathbf{c}_i\|_2$
  until Convergence
  $X_{i+1} = X_i - \mathbf{t}_i \mathbf{t}_i^T X_i$
  $Y_{i+1} = Y_i - \mathbf{t}_i \mathbf{t}_i^T Y_i$
end for
Output: W, T, C, U
