# Supplementary material for: Prediction of clinical depression scores and detection of changes in whole-brain using resting-state functional MRI data with partial least squares regression
Source: PLoS One. 2017 Jul 12;12(7):e0179638. doi: 10.1371/journal.pone.0179638 (PMC5507488; doi:10.1371/journal.pone.0179638)
Supplement: S1 Table — Bold figures represent the best achievement. (PDF) [file pone.0179638.s002.pdf]

Supporting Information

S1 Table. Root mean squared errors in output-age. Bold figures represent the best achievement.

	BDI-II	SHAPS	PANAS(n)	age
OLS	11.6±1.35	7.33±0.81	8.91±0.982	9.89±1.15
PLS	9.71±1.11	6.44±0.77	7.38±0.817	9.40±1.08
KPLS-Poly(2)	**9.56±1.08**	**6.11±0.673**	**7.29±0.807**	9.51±1.09
KPLS-Poly(3)	10.3±1.15	6.43±0.702	7.57±0.831	9.41±1.07
KPLS-Gauss	9.88±1.11	6.49±0.706	7.42±0.821	**9.29±1.05**
