# Supplementary material for: Prediction of clinical depression scores and detection of changes in whole-brain using resting-state functional MRI data with partial least squares regression
Source: PLoS One. 2017 Jul 12;12(7):e0179638. doi: 10.1371/journal.pone.0179638 (PMC5507488; doi:10.1371/journal.pone.0179638)
Supplement: S2 Table — (PDF) [file pone.0179638.s003.pdf]

Supporting Information

S2 Table. Root mean squared errors in input-age.

	BDI-II	SHAPS	PANAS(n)	age
OLS	12.9±1.48	7.90±0.895	9.98±1.11	-
PLS	11.7±1.30	7.52±0.822	8.74±0.962	-
KPLS-Poly(2)	11.7±1.30	7.70±0.842	8.83±0.963	-
KPLS-Poly(3)	12.3±1.36	7.93±0.863	9.21±1.01	-
KPLS-Gauss	10.3±1.17	6.78±0.759	7.69±0.853	-
