# Supplementary material for: Prediction of clinical depression scores and detection of changes in whole-brain using resting-state functional MRI data with partial least squares regression
Source: PLoS One. 2017 Jul 12;12(7):e0179638. doi: 10.1371/journal.pone.0179638 (PMC5507488; doi:10.1371/journal.pone.0179638)
Supplement: S3 Table — (PDF) [file pone.0179638.s004.pdf]

Supporting Information

S3 Table. Root mean squared errors in no-age.

	BDI-II	SHAPS	PANAS(n)	age
OLS	12.5±1.43	7.69±0.868	9.69±1.07	-
PLS	11.4±1.27	7.44±0.804	8.59±0.946	-
KPLS-Poly(2)	11.0±1.22	7.17±0.777	8.24±0.916	-
KPLS-Poly(3)	11.5±1.31	7.32±0.812	8.61±0.973	-
KPLS-Gauss	11.6±1.29	7.87±0.853	8.97±0.971	-
