# Supplementary material for: Prediction of clinical depression scores and detection of changes in whole-brain using resting-state functional MRI data with partial least squares regression
Source: PLoS One. 2017 Jul 12;12(7):e0179638. doi: 10.1371/journal.pone.0179638 (PMC5507488; doi:10.1371/journal.pone.0179638)
Supplement: S4 Table — KPLS-Poly(2) followed by LDA significantly outperformed direct LDA, SVM, and OLS followed by LDA in accuracy (adjusted for multiplicity using the Bonferroni-Holm method with significance level α = 0.05). (PDF) [file pone.0179638.s005.pdf]

Supporting Information

S4 Table. Classification performance. KPLS-Poly(2) followed by LDA significantly outperformed direct LDA, SVM, and OLS followed by LDA in accuracy (adjusted for multiplicity using the Bonferroni-Holm method with significance level $\alpha = 0.05$).

	accuracy (%)	sensitivity (%)	specificity (%)
direct LDA	57.7±4.45	53.4±6.55	61.5±6.03
direct SVM	69.1±4.17	69.0±6.07	69.2±5.72
OLS+LDA	62.6±4.36	62.1±6.37	63.1±5.99
PLS+LDA	72.4±4.03	74.1±5.75	70.8±5.65
KPLS-Poly(2)+LDA	**80.5±3.57**	**81.0±5.15**	**80.0±4.96**
KPLS-Poly(3)+LDA	76.4±3.83	74.1±5.75	78.5±5.10
KPLS-Gauss+LDA	71.5±4.07	70.7±5.98	72.3±5.55
