# Supplementary material for: Prediction of clinical depression scores and detection of changes in whole-brain using resting-state functional MRI data with partial least squares regression
Source: PLoS One. 2017 Jul 12;12(7):e0179638. doi: 10.1371/journal.pone.0179638 (PMC5507488; doi:10.1371/journal.pone.0179638)
Supplement: S5 Table — KPLS-Poly(2) followed by LDA significantly outperformed direct LDA and OLS followed by LDA in accuracy (adjusted for multiplicity using the Bonferroni-Holm method with significance level α = 0.05). (PDF) [file pone.0179638.s006.pdf]

Supporting Information

S5 Table. Classification Performance without subjects over 60. KPLS-Poly(2) followed by LDA significantly outperformed direct LDA and OLS followed by LDA in accuracy (adjusted for multiplicity using the Bonferroni-Holm method with significance level $\alpha = 0.05$).

	accuracy (%)	sensitivity (%)	specificity (%)
direct LDA	66.1±4.27	66.7±6.19	65.6±5.89
direct SVM	67.8±4.21	74.1±5.75	62.3±6.01
OLS+LDA	61.7±4.38	61.1±6.40	62.3±6.01
PLS+LDA	70.4±4.11	72.2±5.88	68.9±5.74
KPLS-Poly(2)+LDA	**78.3±3.72**	**77.1±5.29**	**80.0±5.22**
KPLS-Poly(3)+LDA	73.9±3.96	68.5±6.10	78.7±5.08
KPLS-Gauss+LDA	70.4±4.11	68.5±6.10	72.1±5.56
