# Supplementary material for: Prediction of clinical depression scores and detection of changes in whole-brain using resting-state functional MRI data with partial least squares regression
Source: PLoS One. 2017 Jul 12;12(7):e0179638. doi: 10.1371/journal.pone.0179638 (PMC5507488; doi:10.1371/journal.pone.0179638)
Supplement: S6 Table — Performance with the standard AAL outperformed extended AAL significantly. (adjusted for multiplicity using the Bonferroni-Holm method with significance level α = 0.05). (PDF) [file pone.0179638.s007.pdf]

Supporting Information

S6 Table. Root mean squared errors in extended AAL. Performance with the standard AAL outperformed extended AAL significantly. (adjusted for multiplicity using the Bonferroni-Holm method with significance level $\alpha = 0.05$).

	BDI-II	SHAPS	PANAS(n)	age
OLS	14.0±1.54	9.20±1.00	10.2±1.09	11.5±1.33
PLS	11.9±1.30	7.52±0.832	8.64±0.936	10.4±1.16
KPLS-Poly(2)	12.5±1.34	7.90±0.859	9.58±1.02	10.8±1.20
KPLS-Poly(3)	11.3±1.23	7.25±0.797	8.64±0.927	10.7±1.17
KPLS-Gauss	11.3±1.26	7.28±0.815	8.28±0.906	10.0±1.12
